# Bacterial but no SARS-CoV-2 contamination after terminal disinfection of tertiary care intensive care units treating COVID-19 patients

**DOI:** 10.1186/s13756-021-00885-z

**Published:** 2021-01-12

**Authors:** Daniel A. Hofmaenner, Pedro David Wendel Garcia, Branko Duvnjak, Bhavya Chakrakodi, Julian D. Maier, Michael Huber, Jon Huder, Aline Wolfensberger, Peter W. Schreiber, Reto A. Schuepbach, Annelies S. Zinkernagel, Philipp K. Buehler, Silvio D. Brugger, for the COVID-19 ICU-Research Group Zurich, Jan Bartussek, Phillip Buehler, Dorothea Monika Heuberger, Matthias Peter Hilty, Daniel Andrea Hofmänner, Martina Maibach, Schuepbach Reto Andreas, Pedro David Wendel Garcia

**Affiliations:** 1 Institute of Intensive Care, University Hospital Zurich, Raemistrasse 100, CH-8091 Zurich, Switzerland; 2 Department of Infectious Diseases and Hospital Epidemiology, University Hospital Zurich, Raemistrasse 100, CH-8091 Zurich, Switzerland; 3 Institute of Medical Virology, University of Zurich, Winterthurerstrasse 190, CH-8057 Zurich, Switzerland

**Keywords:** SARS-CoV-2, Disinfection, Hydrogen peroxide nebulisation, ICU, Hospital epidemiology, Nosocomial infection

## Abstract

**Background:**

In intensive care units (ICUs) treating patients with Coronavirus disease 2019 (COVID-19) invasive ventilation poses a high risk for aerosol and droplet formation. Surface contamination of severe acute respiratory syndrome Coronavirus 2 (SARS-CoV-2) or bacteria can result in nosocomial transmission.

**Methods:**

Two tertiary care COVID-19 intensive care units treating 53 patients for 870 patient days were sampled after terminal cleaning and preparation for regular use to treat non-COVID-19 patients.

**Results:**

A total of 176 swabs were sampled of defined locations covering both ICUs. No SARS-CoV-2 ribonucleic acid (RNA) was detected. Gram-negative bacterial contamination was mainly linked to sinks and siphons. Skin flora was isolated from most swabbed areas and *Enterococcus faecium* was detected on two keyboards.

**Conclusions:**

After basic cleaning with standard disinfection measures no remaining SARS-CoV-2 RNA was detected. Bacterial contamination was low and mainly localised in sinks and siphons.

## Background

During the last months, the novel Coronavirus disease (COVID-19) pandemic has challenged many health-care systems worldwide. In line with the growing knowledge about specific pathophysiologic mechanisms, COVID-19 has increasingly been associated with bacterial co-infections, exhibiting high rates in intensive care units (ICUs) [1–4]. It is estimated that around 50% of fatalities associated with COVID-19 had secondary infections contributing to the disease course with a relevant impact on patient outcomes [5, 6]. Treating critically ill COVID-19 patients often involves mechanical ventilation, which poses a high risk for droplet and aerosol transmission. In addition to the risk of severe acute respiratory syndrome Coronavirus 2 (SARS-CoV-2) contamination of surfaces and medical equipment, bacterial pathogens complicating the disease course can be transmitted to the environment surrounding the patient. Equipment and surfaces (e.g. respirators, beds) are well known for contributing to the transmission of bacterial pathogens promoting nosocomial infections [7, 8].

With the decline of the current COVID-19 outbreak in some countries, affected ICUs now returning to treating non-COVID-19 patients are being faced with the challenge of disinfecting their equipment and facilities in order to prevent hospital-acquired infections. Moreover, it is currently not well characterised whether or how long SARS-CoV-2 ribonucleic acid (RNA) persists on surfaces after complete disinfection of ICUs.

The aim of this study was to investigate the burden of pathogenic bacteria and SARS-CoV-2 RNA on different devices and surfaces after terminal cleaning measures in two specialised ICUs of a tertiary care hospital exclusively treating COVID-19 patients.

## Methods

### Study setting

This single-blinded study was conducted at the University Hospital Zurich, Switzerland, a tertiary care hospital with around 980 beds. During the COVID-19 pandemic, two defined subunits of the ICU were designated to exclusively treat COVID-19 patients. The first (ICU 1) had the capacity to treat 16 patients, the second (ICU 2) 12 patients. In total, ICU 1 treated 39 COVID-19 patients (661 patient days). ICU 2 treated 14 COVID-19 patients (209 patient days). No institutional review board (IRB) approval was required as this study did not involve any patients.

### ICU cleaning/disinfection

After the first COVID-19 wave, both subunits were cleaned in order to provide care for non-COVID-19 patients. All surfaces, devices and floors in ICU 1 were disinfected by nurses and cleaning staff with aldehyde-based Kohrsolin^®^ 1% (Wilbert Hygiene, Germany). In addition, a dry mist hydrogen peroxide (H_2_O_2_) nebulisation technique (Dosymist XL, SolidFog Technologies^®^, Belgium and H_2_O_2_ à 12% Fisher Scientific^®^, USA) was used in ICU 1 in the ICU pharmacy room. The purpose to use this relatively novel technique in the present study was to gain more experience with it and to test it in a small, designated area. With the aid of the hydrogen peroxide technology, a dry aerosol is generated, with the oxidative action of hydroxyl radicals leading to antimicrobial properties. Quality control of the H_2_O_2_ disinfection was performed with chemical indicators (3M Healthcare^®^, USA) [successful decontamination indicated by color change on test kit] and biological indicators (*Geobacillus stearothermophilus*, Crosstex^®^, USA) [successful decontamination indicated by 6-log reduction between positive and negative control]. Surfaces, devices and floors in ICU 2 were disinfected by nurses and cleaning staff with Kohrsolin^®^ 1% (Wilbert Hygiene, Germany) only. To minimize biases, nurses and cleaning staff were not aware that this study would be conducted in both ICUs. Cleaning and disinfection were not supervised by the study team.

### Virus sampling and analysis

**Fig. 1:**
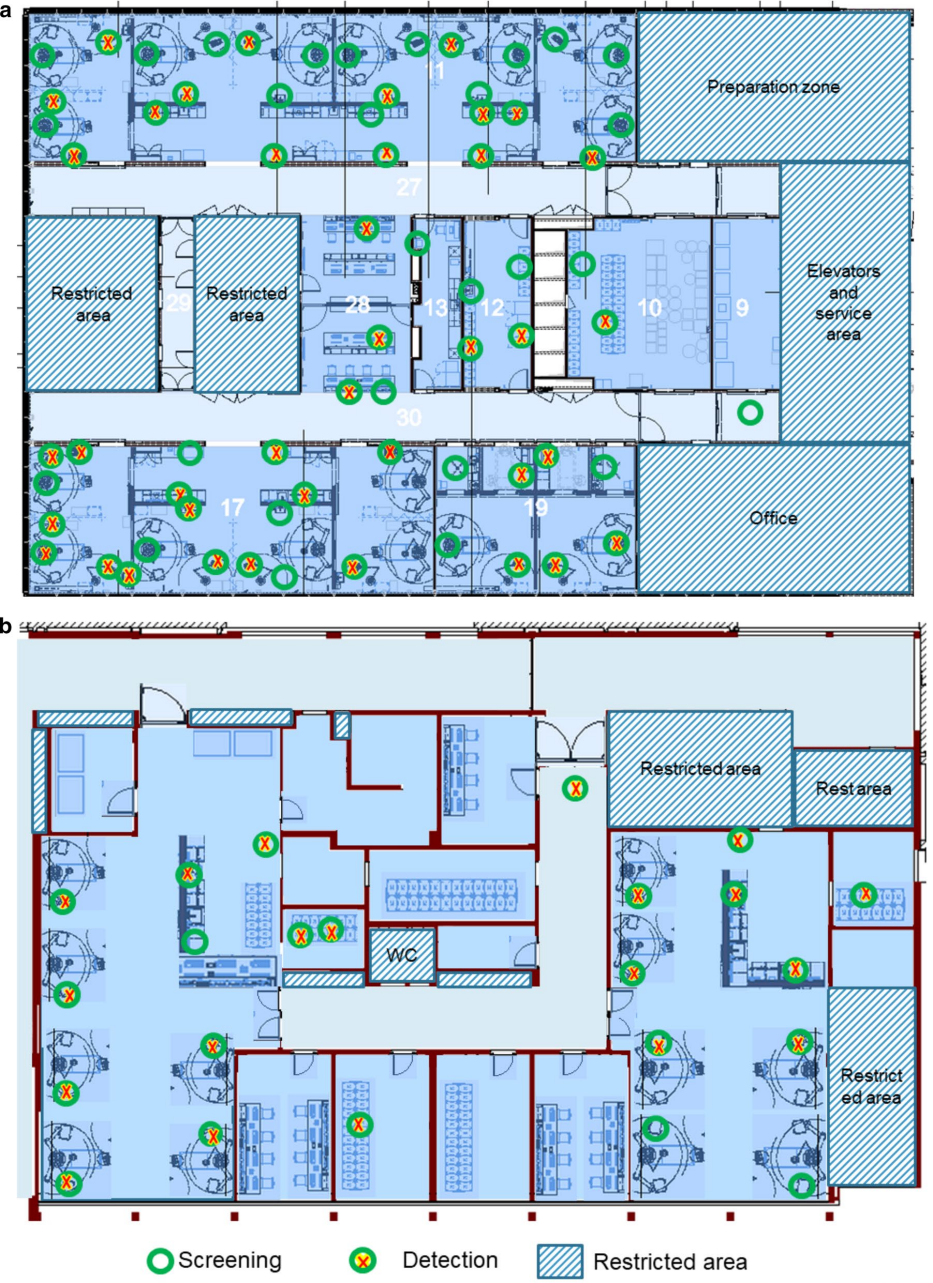
Both ICU subunits with indicated sampling points. **a** represents ICU 1, **b** represents ICU 2

After the cleaning procedures, 86 viral swabs (FLOQSwabs, UTM-RT transport medium, Copan^®^, Italy) were taken from different locations in both ICUs (representing high-frequency touch surfaces, patient-surrounding areas, healthcare-worker changing areas and sinks including siphons) with a minimal swabbing surface dimension of 10 × 10 cm. Sampling objects were 30 monitors/keyboards (bedside), 8 tabletops to prepare drugs for application including adjacent sinks, 17 individual sinks including siphons in patient rooms, 6 floors of patient compartments and 1 main exit gate (floor), 13 respirator touch screens and handling buttons, 2 physician’s work desks, 2 non-personalised telephones, 2 perfusors, 4 rolling trolleys/tables, 2 fridge doors in the ICU pharmacy, 1 chair and 2 surfaces in kitchens. Due to the different configurations and designs of the two ICU subunits, it was not possible to take an equal amount of swabs from both ICU subunits. Figure 1 demonstrates both ICU subunits and sampling points are indicated.

The swabbed area was individualised according to the swabbed object size and shape. Real-time reverse transcriptase polymerase chain reaction (rtPCR) was performed to detect SARS-CoV-2 RNA with inclusion of positive and negative control. PCR conditions were as previously described by Corman et al. [9].

### Bacterial sampling, culture, identification and resistance testing

In parallel to the viral sampling, a total of 90 moistured swabs (eSwabs™, Copan^®^, Italy, comprising 1 millilitre of Amies medium) were taken from the same locations in both ICUs, analogous to the viral sampling (Fig. 1).

Dependent on the object size and shape, the swabbed area was individualised. In general, the largest possible area to swab was chosen.

All swabs were plated on 5% sheep blood agar and MacConkey agar plates in three fractions. Subsequently, the samples were incubated for 48 h at 37 °C in ambient air. After incubation, colony forming units (CFU) were analysed semiquantitatively. Growth in the first fraction of the agar plate corresponded to 10^3^–10^4^ CFU/ml, in the first and second fraction to 10^4^–10^5^ CFU/ml and in all three fractions to 10^5^–10^6^ CFU/ml.

Subcultures were plated on 5% sheep blood agar or MacConkey agar and identification was done as previously described [10] with the exception that identification was done using both a BD Phoenix™, Becton Dickinson, USA, automated identification and susceptibility testing system as well as a Bruker MALDI-TOF–MS biotyper (Bruker, Germany). Susceptibility testing was done using the BD Phoenix system.

## Results

All of the 86 viral swabs sampled tested negative for SARS-CoV-2 RNA. Two samples from ICU 1 (sink in the pharmacy and one monitor/keyboard) were initially weakly positive but inconclusive. The quantification cycle values for those two swabs were 43.17 and 38.94 respectively. However, this finding could not be reproduced in a subsequent confirmatory test.

All chemical and biological indicators demonstrated successful (bacterial) decontamination of the ICU 1 pharmacy room. Tables 1 and 2 summarise cultivated bacteria on different surfaces of both subunits of the ICU after disinfection. In ICU 1, most bacteria were found in the sinks and associated siphons—in the individual sinks in patient rooms and at tabletops employed for drug preparation by health care staffing (Table 1). *Pseudomonas aeruginosa, Pseudomonas putida, Stenotrophomonas maltophilia, Klebsiella oxytoca and Enterobacter cloacae* were cultured from these areas. In line with ICU 1, *Pseudomonas aeruginosa, Pseudomonas putida, Stenotrophomonas maltophilia and Achromobacter* spp. were found in ICU 2 in the sinks (Table 2). No multidrug-resistant (MDR) *P. aeruginosa* was detected.

**Table 1:** Bacterial contamination on ICU 1 according to swabbed objects and swab count. Numbers in () indicating the fraction of positive samples compared to the total number of swabs per object group

	Number of swabs from distinct objects (n = 67)	*Pseudomonas* spp.	Other detected bacteria
*Bacterial contamination ICU 1*			
Monitors/keyboards	18		Coagulase-negative staphylococci 10^3^–10^4^/ml (10/18)
Tabletops, sinks and siphons in drug preparation areas	5	*Pseudomonas aeruginosa* 10^5^–10^6^/ml (2/5), *Pseudomonas putida* 10^5^–10^6^/ml (1/5)	*Stenotrophomonas maltophilia* 10^5^–10^6^/ml (3/5), *Enterobacter cloacae* 10^5^–10^6^/ml (1/5), coagulase-negative staphylococci 10^3^–10^4^/ml (1/5)
Individual sinks in patient area	12	*Pseudomonas aeruginosa* 10^5^–10^6^/ml (2/12), *Pseudomonas* spp. 10^3^–10^4^/ml (1/12)	*Klebsiella oxytoca* 10^4^–10^5^/ml (1/12), *Stenotrophomonas maltophilia* 10^5^–10^6^/ml (1/12), coagulase-negative staphylococci 10^3^–10^4^/ml (1/12)
Floors in patient compartment	6		Coagulase-negative staphylococci 10^3^–10^4^/ml (3/6)
Respirators	13		Coagulase-negative staphylococci 10^3^–10^4^/ml (1/13)
Work desk physicians	2		Coagulase-negative staphylococci 10^3^–10^4^/ml (2/2)
Telephones non-personalized	2		Coagulase-negative staphylococci 10^3^–10^4^/ml (1/2)
Perfusors	2		
Rolling trolleys/tables	4		Coagulase-negative staphylococci 10^3^–10^4^/ml (2/4)
Fridges pharmacy	2		
Chair	1

**Table 2:** Bacterial contamination on ICU 2 according to swabbed objects and swab count. Numbers in () indicating the fraction of positive samples compared to the total number of swabs per object group

	Number of swabs from distinct objects (n = 23)	*Pseudomonas* spp.	Other detected bacteria
*Bacterial contamination ICU 2*			
Monitors/keyboards	12		*Enterococcus faecium* 10^3^–10^4^/ml (2/12), coagulase-negative staphylococci 10^3^–10^4^/ml (8/12)
Tabletops, sinks and siphons in drug preparation areas	3		Coagulase-negative staphylococci 10^3^–10^4^/ml (3/3)
Individual sinks in patient area	5	*Pseudomonas aeruginosa* 10^4^–10^5^/ml (1/5), *Pseudomonas putida* 10^4^–10^5^/ml (1/5), *Pseudomonas* spp. 10^4^–10^5^/ml (2/5)	*Achromobacter* 10^3^–10^4^/ml (1/5), *Stenotrophomonas maltophilia* 10^4^–10^5^/ml (1/5), coagulase-negative staphylococci 10^4^–10^5^/ml (2/5)
Floor main exit gate	1		Coagulase-negative staphylococci 10^3^–10^4^/ml (1/1)
Surface patient kitchen	2		Coagulase-negative staphylococci 10^3^–10^4^/ml (1/2)

*Enterococcus faecium* was cultivated in two out of 12 swabs from monitors/keyboards. In both ICUs, coagulase-negative Staphylococci were found in most swabbed areas but with low CFU numbers (mostly 10^3^–10^4^ per ml corresponding to less than 5 CFU/cm^2^) (Tables 1 and 2).

## Discussion

The aim of this study was to investigate the persistence of SARS-CoV-2 and bacteria on different devices and surfaces after disinfecting measures in two tertiary care ICU subunits that had exclusively treated COVID-19 patients. The main finding of this study is that no SARS-CoV-2 RNA was detected by rtPCR. However, pathogenic bacteria were abounded mainly in sinks and corresponding siphons of the ICUs and enterococci were isolated from two monitors. Gram-positive bacteria were mainly isolated from surfaces (e.g. monitors/keyboards) whereas Gram-negative bacteria were mainly detected in plumbing units.

To date, it has not been clearly established how long and to what extent SARS-CoV-2 RNA contaminates environmental surfaces and devices in hospitals. A recent study has described a low percentage of positive samples in an emergency room and the sub-intensive care ward [11]. In another study, almost 57% of rooms treating COVID-19 patients had at least one environmental surface contaminated [12].

In our study, viral RNA was not detectable on any of the swabbed locations of the two ICU subunits, which suggests that conventional disinfection measures are likely sufficient to prevent further spread of the virus. This finding thus might contribute to infection and prevention policies.

Already back in 1974, The Lancet published an article describing isolation of *Pseudomonas aeruginosa* in hospital sinks [13]. Since then, various studies have investigated hospital water supply systems as potential reservoirs for the transmission of pathogenic bacteria in hospitals [14–18].

In line with previous studies [15, 16, 18], we found *Pseudomonas aeruginosa* in sinks/siphons in both ICUs. Specifically regarding the ICU setting, Zhou et al. demonstrated that sink traps may act as an important source of *Pseudomonas* spp. leading to colonisation or infection of critically ill patients [18]. Additionally, stagnant water in general bears a risk for being a reservoir and source of outbreaks with several pathogens [19]. This finding highlights the need for constant awareness of possible bacterial reservoirs and the need for further refining cleaning methods in the ICU.

In this line of research it has been suggested that devices applying heat and electromechanical vibration to siphons lead to lower colonisation of patients with multidrug-resistant (MDR) *Pseudomonas spp.* [16] and that self-disinfecting sinks reduced *Pseudomonas spp.* bioburden in a pediatric ICU [20], offering possibilities to lower transmission of MDR pathogens*.* Whether such measures prove feasible and effective in other ICU settings remains to be elucidated in further studies and is beyond the scope of this study. However, no MDR *Pseudomonas aeruginosa* was detected in our ICUs despite the fact that several patients in the ICU had been colonised with MDR *Pseudomonas aeruginosa*.

Other cultured bacteria associated with moist environments were *Stenotrophomonas maltophilia, Klebsiella oxytoca and Achromobacter* spp., all Gram-negative, potentially harmful bacteria. *Stenotrophomonas maltophilia* is known to cause hospital-acquired infections and is associated with multidrug resistance [21, 22]. *Klebsiella* spp. can be responsible for infectious complications, especially hospital-acquired pneumonias, urinary tract infections or septicemias [23]. Furthermore, *Achromobacter* spp. have been linked to increased risk of morbidity and mortality in critically ill patients in a study reporting a 18 month lasting epidemic in an ICU [24]. Additionally, *Enterobacter cloacae* was cultured from sinks located next to tabletops employed for drug preparation in ICU 1. Enterobacterales (especially carbapenem-resistant strains) are an important cause of healthcare-associated infections and transmission by means of environmental reservoirs, such as therapeutic beds, has been described [25, 26]. The colonisation of critically ill patients with carbapenemase-producing Enterobacterales has been associated with an increased length of stay, as well as with a 1.8 higher death hazard ratio as opposed to non-colonised ICU patients [27].

Such findings mandate thorough disinfection of ICUs and surveillance of sinks and siphons as a source for further outbreaks. Water-free ICUs might reduce the risk of nosocomial transmission from bacteria residing in sinks and siphons [28].

Monitors and keyboards are frequently used devices in the everyday care in the ICU. We found *Enterococcus faecium* on two out of 12 monitors/keyboards in ICU 2, a pathogen known to cause hospital-acquired infections and possibly exhibiting complex resistance patterns, e.g. vancomycin-resistant strains. The failure of disinfection in this case is most likely because of the applied manual disinfection, which is prone to failure. However, we were not able to assess the individual cleaning performance. In a large multicenter, randomised trial, a cleaning bundle (REACH) showed promising results in reducing especially enterococcal infections [29]. The implementation of such bundles in ICUs might probably lead to less nosocomial infections in critically ill patients and warrants investigation in further trials.

Coagulase-negative staphylococci were detected in the majority of the swabbed locations. These bacteria are known to be associated with human skin and mucosa and can be a source for infections of foreign bodies or indwelling catheters [30]. It remains speculative, whether the finding of these bacteria in the disinfected ICUs has a clinical relevance with regard to the transmission of potentially harmful infections.

The use of the hydrogen peroxide nebulisation technique has been shown to reduce bacterial burden on medical devices [31]. In the present study, we did not analyse the effectiveness of this technique as compared to control locations without its use. However, we were not able to culture bacteria in the areas where hydrogen peroxide nebulisation was used. This supports the possible role of hydrogen peroxide as a useful adjunct for ICU disinfection. Another advantage of this technique is the operator-independence.

Strengths of this study are the structured and comprehensive viral/bacterial sampling of different objects and its pragmatic design during COVID-19 pandemics reflecting everyday situations in ICUs with possibly relevant impacts on patient outcomes. Furthermore, the single-blinded study design ensured the investigation of regular cleaning quality.

Our study has several limitations. First, the ICUs were cleaned and disinfected by numerous employees of the ICU department and facility management. As a consequence, different parts of the facilities were probably not cleaned uniformly or in the same intensity. However, this fact reflects daily real-life work processes in hospitals. Second, the investigators could not verify if the objects actually had been cleaned or had been omitted owing to human factors such as overlooking or ignoring.

Third, the analysed objects differed in shape and size, which might have influenced the swabbing technique. Fourth, no cluster-randomisation could be performed between the two ICU subunits due to time-constraints and urgent demand for ICU beds. Finally, due to the dynamic of the pandemic in our hospital, both ICUs were not swabbed and tested as a baseline prior to cleaning and disinfection and the timing of the swabbing was not randomly performed. This should be taken into account in future studies with a similar design.

## Conclusion

In conclusion, several bacteria possibly leading to colonisation or infections of critically ill patients were cultured from swabs taken from disinfected ICUs after the COVID-19 pandemic. SARS-CoV-2 RNA was not detected on any of the swabbed objects further supporting that conventional disinfection is sufficient to safely repurpose COVID-19 ICUs to routine ICUs despite the contagious nature of the disease.

## Data Availability

The datasets used and analysed during the current study are available from the corresponding author on request.

## Abbreviations

ICU: Intensive care unit
SARS-CoV-2: Severe acute respiratory syndrome Coronavirus 2
RNA: Ribonucleic acid
COVID-19: Coronavirus disease 2019
IRB: Institutional review board
H_2_O_2_: Hydrogen peroxide
rtPCR: Real-time reverse transcriptase polymerase chain reaction
CFU: Colony forming units
MDR: Multidrug-resistant
